# On the Theories of Animal Heat

**Published:** 1853-10

**Authors:** B. Brodie


### SELECTIONS

FROM

### FOREIGN AND HOME MEDICAL JOURNALS.

---

*On the Theories of Animal Heat.*—By Sir B. BRODIE, Bart.—The general evidence in favor of the chemical theory of animal heat has received many important additions of late years. The fact that a considerable amount of heat is generated by plants during the processes of germination and flowering, when, like animals, they withdraw a large amount of atmospheric oxygen, and replace it by carbonic acid set free from their own tissues, has long been known; and very careful observations upon the *Colocasia odora* a plant of the Arum family, which is remarkable for the elevation of temperature which its spadix undergoes at the height of the flowering period, have demonstrated that the production of heat is constantly accompanied by the generation of carbonic acid, that it does not take place if the flowers be made to expand in an atmosphere of nitrogen, and that during both the attainment of the highest point and the subsequent decline, there is a close correspondence between the amount of oxygen consumed in successive hours, and the excess of heat shown by the spadix above that of the surrounding medium. So, again, the researches of Mr. Newport upon the insect temperature have shown that the same constant coïncidence exists between that generation of heat which they occasionally exhibit, and the replacement of atmospheric oxygen by carbonic acid. Moreover, since we may regard it as all but demonstrated that the animal can under no circumstances convert non-azotized saccharine or oleaginous compounds so abundantly supplied by plants into the albuminous constituents of its own tissues, the large amount of these substances consumed by animals, and the provision obviously made for their digestion and for their reception into the circulation become utterly meaningless, if they are not introduced for the express purpose of being applied to the maintenance of the combustive process, and the consequent generation of heat. Further, the direct effect of withholding food altogether, as the experiments of Chossât have shown, is to produce a complete absorption of the fat, which, with the products of the waste of the azotized tissues, is burned off day by day for the maintenance of the animal temperature; the duration of life in this condition is strictly proportional to the amount of combustive material previously stored up in the form of adipose tissue; and when this is all exhausted, the heat of the body, which had previously suffered but little decline, diminishes from hour to hour, until the animal dies of cold — the application of external warmth being still able to arouse it to activity, even when it has sunk into a state of torpor approaching to coma. All experiments upon the respiration, moreover, concur in showing that the liberation of carbonic acid is much more rapid at low temperatures than at high; showing that there exists within the system some means of self-adjustment, whereby the amount of fuel consumed is proportioned to the quantity of heat which it requires to produce, for the maintenance of its temperature at the regular standard.

The recent researches of M. Bernard upon the functions of the liver, leave but little room for doubt that one of the most important purposes of this organ is to prepare the respiratory pabulum; not so much, however, in the mode originally supposed by Liebig, by separating it from the blood as bile, this to be reäbsorbed and subsequently burned off in the lungs; but rather by transforming, without separation, certain materials previously contained in the blood, so that as the current passes through this gland, both the substances newly absorbed from the alimentary canal, and those which have been taken up in the course of the general circulation, are subjected to its influence, and then pass on, through the right cavities of the heart, to the pulmonary vessels, in a state of peculiar fitness to undergo a further transmission by the influence of the oxygenated atmosphere which they there encounter. The combustive materials directly supplied by the food are chiefly, in herbivorous animals, those of the farinaceous and saccharine, and those of the oleaginous types. The farinaceous matters are converted by the digestive process into the saccharine form ; so that grape-sugar is found in the mesenteric veins after the ingestion of such aliment, as is also cane-sugar if this have been taken in. But the blood is not very tolerant of the presence of grape-sugar, still less of that of cane-sugar; so that if these substances be injected into the *jugular vein* in any quantity, they are speedily detectible in the urine. During their passage through the liver, however, they are converted Into a new form of sugar, which has been termed "liver-sugar," the characteristic properties of which seem to consist in its differing from grape-sugar, as grape-sugar differs from cane-sugar, that is, in being far more tolerated by the blood, and in being more readily converted into lactic acid. Hence when grape-sugar and cane-sugar are injected into the *vena portæ*, they do not show themselves in the urine ; and the liver-sugar which is produced by their transformation is not traceable further than the pulmonary vessels, being speedily transformed, and in a great part eliminated (probably passing the intermediate stage of lactic acid) by the respiratory process. But it is one of the most curious results of M. Bernard's researches, that the liver not only thus transforms other saccharine compounds, but that it generates sugar *de novo*, under circumstances which seem to forbid our regarding it as anything else than a product of the retrograde metamorphosis of the albuminous compounds. For M. Bernard has found sugar in the liver and hepatic blood of carniverous animals, and even in the liver of the mammalian fœtus and of the embryo chick ; and so constant does the presence of this substance appear to be, that the production of a peculiar kind of sugar, specially fitted for undergoing the combustive process, must henceforth be regarded as one of the most important functions of the liver. It has been supposed that the sugar generated by the liver may be derived from fatty matters brought to it by the vena portæ; but we cannot find in Bernard's experiments any evidence of this; and whilst we have no chemical reason for believing that sugar can be formed by the transformation of fat (which would be an inversion of the usual order of metamorphosis) we know that it may be generated as one of the products of the disintegration of muscular tissue, the juice of flesh having yielded *inosite*, a new form of sugar, to the analysis of Scherer.

It has been further shown by M. Bernard, that the liver produces a peculiar fat, from the constituents of the blood which passes through it; and it seems that this liver-fat, like the liver-sugar, may be the product of the transforming process, exercised alike upon materials newly absorbed from the alimentary canal, and upon the products of the disintegration of the tissues. That albuminous compounds in a state of retrograde metamorphosis may generate fatty matter can scarcely, we think, be any longer questioned; since the phenomena of fatty degeneration concur with chemical evidence, to prove that this is one of the forms into which their elements pass, in preparation for their egress from the body.

Thus, then, it appears that the *respiratory pabulum* of carniverous animals, which is derived from the decomposition of the albuminous constituents of their food and of their own tissues, precisely corresponds with that which is furnished to the herbivorous races in the non-azotized articles of their diet; that the liver may be regarded as an organ specially destined to alter the characters of the blood, not merely by what it separates *from* it, but also by the changes it makes *in* it; and that these changes have reference to the constant sustenation of the combustive process, by the preparation of certain organic compounds peculiar to the living body, of a nature that may enable them readily to undergo oxidation when exposed to the air. And it is a most interesting and suggestive fact, that the products of the disintegration of the system should thus be applied—by passing through the very same course—to the same final uses, as are served in a large proportion of the animal kingdom by organic compounds that cannot be rendered subservient to the production of animal tissue. We can scarcely fail to see in the whole of this arrangement, not merely a means of freeing the system from effete matters, but a scheme for the regular supply of the combustive apparatus with appropriate fuel; and those, if such there be, who deny that the production of animal heat is essentially dependent upon the conversion of hydrocarbonaceous compounds into water and carbonic acid have to explain how these compounds can be eliminated, as we know they are by means of this exidating process, without the production of heat; and they have also to explain why the herbivorous animal should be instinctively impelled to injest a large amount of food, that can serve no histogenetic purpose in the economy, and why their digestive apparatus should be specially adapted to the reduction, solution, and conversion of non-azotized compounds, as well as to the introduction of azotized matter into the system.

The recent researches of M. Barrall "On the Statistics of the Human Body," have afforded much additional information upon the subject of our present inquiry. He has carefully determined the ultimate constituents of the food, and of the various excretions, of four individuals, during five days, experimenting thus upon himself also during summer as well as in the winter; and presents us the results in an elaborate series of tables. We shall cite only a few of the most important. In the first place, the *carbon* of his food amounted to 5654.1 grains in winter, and 4090 grains in summer; and of this the proportion ejected in the form of *feces* was 236.2 grains in winter, and 139.4 in summer; that eliminated by the *urine* was 234.6 grains in winter, and 211.5 grains in summer; whilst that which passed off by exhalation (pulmonary and cutaneous, the latter, however being an insignificant fraction) was no less than 5183,3 grains in winter, and 3741.1 in summer. Thus we see how large a proportion of the carbon of the food is thrown upon the respiratory process for elimination, and how considerable a diminution takes place in the amount exhaled during the heat of summer, involving a corresponding difference in the demand for aliment. M. Barral's researches appear to afford the means of determining approximately at least, how much of the *hydrogen* of the food is oxidized in the respiratory process, and how much of the water exhaled is really generated combustively. He found that of the whole amount of hydrogen taken in, not more than from one-eighth to one-tenth passes off by the other excretions, the remaining seven-eighths or nine-tenths being exhaled in the condition of watery vapor by the lungs. Now of the *oxygen* ingested, he found that 3841.4 grains in winter, and 2757.6 grains in the summer, passed off by the lungs in the condition of water; and this would, of course, carry off its equivalent of hydrogen, also derived from the food, without any production of heat.* But the amount of hydrogen actually exhaled was 801.3 grains in winter, and 597.5 grains in summer; so that after deducting one-eighth of the weight of the oxygen, there remain 321.1 grains in winter, and 252.8 grains in summer, as the amount of hydrogen which could not have been oxidized and eliminated as water, save at the expense of the oxygen of the air, and which must therefore have undergone a combustive process whereby heat could not but have been generated.

Thus it appears certain, that we are not to look only at the combustion of carbon but to that of hydrogen, as a source of animal heat; but it is scarcely fair to reckon (as Prof. Liebig has done) the whole surplus of the oxygen absorbed, above that contained in the carbonic acid exhaled, as being consumed in this mode. For there are other oxidizing processes going on within the system, some of them of no mean importance; more especially the production of the sulphuric and phosphoric† acids

---

* This will be obvious enough if we take the simple case of sugar, which is carbon plus the components of water, already united in equivalent proportions; the carbon being eliminated by combustion the water remains, to be carried off as such. A fatty body, on the other hand, is carbon plus hydrogen plus the components of water; here a portion of the hydrogen, as well as the whole of the carbon, is destitute of its equivalent of oxygen, and water is actually generated in the combustive process, in addition to that whose components were already united in the substance, although under another form.

† Professor Liebig has lately asserted, with his usual dogmatism, that no phosphorns exists in the body, or in the alimentary materials, except in the state of phosphoric acid; and further, that the notion that such other compounds of phosphorus exist in the body, and that phosphorus occurs in the protein-compounds in a state analogous to that in which from the sulphur and phosphorus contained in the protein compounds of the food, giving rise to the production of the alkaline sulphates and phosphates which are excreted by the urine. In fact, it may probably be stated, that there is scarcely a single chemico-vital transformation in the body, either *progressive* (that is to say, tending to the formation of living tissues) or *retrograde* (that is, tending to the reduction of the more complex histogenetic substances to the comparatively simple compounds which present themselves in the excretions) in which oxygen is not concerned. Thus, then, although the production of carbonic acid and water may be immediately affected by the combustive metamorphosis of saccharine and fatty matters, and although these matters, when not directly supplied as such by the food, are prepared within the body for the respiratory operation, yet the process of such preparation is one that really involves a great number of separate stages of metamorphosis, beginning with the conversion of alimentary materials into living tissue, and including all the changes which take place during the life of that tissue, and in the progress of its final disintegration. Hence, although the production of such part of the carbonic acid aud water, as proceeds directly from the union of the carbon and hydrogen of the alimentary materials with the atmospheric oxygen, may be looked on as a simple combustive operation, yet as to the whole of the remainder, which proceeds from the disintegration of the tissues themselves, it must be admitted that the ap-

---

sulphur exists in them, "proceeds generally from amateurs in science, and rests on superficial observations, without the slightest scientific foundation." See his "Familiar Letters," third edition, pp. 451–2." We apprehend that several chemists, of no inferior authority as analysts, maintain a different opinion; and that there is abundant physiological evidence derived from the occasional lumiuosity of the urine, breath, and sweat, as well as of the solids of the body when nndergoing decomposition after death, or even before life is extinct, which proves the existence ot phosphorus either in a basic or in an imperfectly oxidized condition, as one of the normal constituents of the body; this phosphorus being usually completely oxidized within the system, and carried off as a phosphoric acid in combination with alkaline bases, but passing into the excretions in its lower state, when, either from an absolute deficiency o respiration, or from the greater demand for oxygen set up by other matters contained in the blood (alcohol, for example) it does not undergo complete metamorphosis.

pearance of a given quantity of carbonic acid in the expired air, can only be looked on as the exponent or manifestation of a long series of changes of composition taking place in the interior of the body, of which it is one of the ultimate products. Some of the advocates of the Chemical Theory of Animal Heat, have spoken of the body as if it was a mere furnace, into which a certain quantity of fuel is put, whose combustion affords a given equivalent of heat; whilst from its chimney there proceed so much carbonic acid, so much water, and so much imperfectly consumed smoke. The whole series of intermediate operations, whose original materials and final products alone are thus recognised, is completely ignored; and the confident assertions of the chemist, based, as they appeared to be, upon a very broad foundation in fact, have passed muster with the general, and even with the medical public, notwithstanding that there is a considerable body of facts, which are not only not explained by chemical doctrine, but which seem, at first view, to be really in antagonism with it. These facts cannot be passed over, either by the scientific physiologist or by the observant practitioner; for they enforce themselves upon the attention of the latter; whilst they must be recognised by the former, as having no less a title to his consideration than have the ordinary phenomena of calorification;—we refer to the local development of heat, or to local deficiency, altogether irrespective of the activity of the circulation; and to the connexion of such irregularities with peculiar conditions of the nervous system.—*Brit. and For. Med. Chir. Review*

